# Acute hepatitis B virus infection in pregnancy: a case report of three patients with comorbid anxiety and their clinical outcomes

**DOI:** 10.3389/fpsyt.2025.1695165

**Published:** 2025-10-22

**Authors:** Shenlu Wu, Kejie Hu, Yufang Wang

**Affiliations:** ^1^ The Second School of Clinical Medicine, Zhejiang Chinese Medical University, Hangzhou, Zhejiang, China; ^2^ Department of Gastroenterology, Hangzhou Third People’s Hospital, Hangzhou, Zhejiang, China

**Keywords:** acute hepatitis B, perinatal mental health, virological clearance, early gestation, case report

## Abstract

This case series reports three pregnant women with acute hepatitis B virus (AHB) infection, exploring their clinical outcomes and comorbid anxiety. All three patients presented with abnormal liver function and positive HBV markers. They received supportive therapy (liver-protective, enzyme-lowering agents, etc.), achieving HBsAg clearance and virological cure, with normal liver function during follow-up (one patient lost to follow-up). Despite favorable biomedical outcomes, all experienced significant anxiety over maternal-fetal health and disease progression. This study emphasizes that AHB in pregnancy, though usually self-limited, requires integrating psychosocial support into multidisciplinary management to address perinatal mental health, supporting WHO’s 2030 viral hepatitis elimination goal.

## Introduction

Despite effective vaccines and antiviral drugs, hepatitis B virus (HBV) continues to pose a major global public health challenge. Aiming to eliminate viral hepatitis as a public health threat by 2030, the World Health Assembly endorsed the Global Health Sector Strategy (GHSS-VH) 2016–2021 in May 2016 (1). The strategy defines elimination as achieving a 65% reduction in mortality and a 90% reduction in incidence (1). Approximately 5-10% of patients with acute hepatitis B (AHB) progress to chronic infection, which may further develop into liver cirrhosis or even hepatocellular carcinoma; a small proportion of patients may also experience acute liver failure (2). Compared with immunocompetent adults, pregnant women are more likely to progress to chronic HBV infection following acute exposure due to pregnancy-associated immunological alterations. However, AHB during pregnancy is relatively uncommon in clinical reports. Studies revealed that maternal hepatitis B surface antigen (HBsAg) carriage was significantly associated with an increased risk of pregnancy-induced hypertension, gestational diabetes mellitus, meconium peritonitis, postpartum hemorrhage, intrahepatic cholestasis, fetal distress, cesarean delivery and macrosomia (3–6). Furthermore, HBV load during the second trimester was identified as an independent risk factor for preterm birth, with each log10 increase in viral load corresponding to an 18% elevation in risk. The risk of preterm delivery was 2.10 times higher in HBV DNA-positive carriers than in their DNA-negative counterparts (7).

Pregnancy is a period of heightened vulnerability to psychological distress, with anxiety disorders affecting approximately 20% of pregnant individuals (8). A meta-analysis found that one in five women in low- and middle-income countries experiences anxiety disorders during pregnancy and the postpartum period (9). Individuals with resolved HBV infection had an elevated risk of depression relative to uninfected individuals (8.5% vs. 10.6%) (10). In another survey, anxiety and depression were reported in 24.5% and 45.9% of individuals with HBV infection, respectively (11). However, little is known about the psychological burden in women with acute HBV infection, as most literature focuses on chronic HBV or non-infectious perinatal anxiety.

Therefore, pregnant women with acute HBV infection are at risk not only of hepatic decompensation but also of significant psychological distress. This report describes three cases of acute HBV infection concurrent with pregnancy. However, despite achieving virological cure, the patient still experienced significant psychological distress. This outcome underscores the imperative need to integrate psychosocial support into comprehensive hepatitis management strategies alongside biomedical care.

## Case report

### Case 1

#### Patient information

A 28-year-old woman was admitted with HBsAg positivity for 6 days and abnormal liver function tests for 1 day. She had undergone hospitalization in the gynecology department for a missed abortion 6 days earlier, during which hepatitis B serology revealed HBsAg >250.00 IU/mL, hepatitis B e antigen (HBeAg) 55.719 S/CO, hepatitis B core antibody (HBcAb) 3.42 S/CO, and IgM antibody against HBcAg (HBcAb-IgM) 0.07 S/CO. At that time, liver function and complete blood count (CBC) were normal. She subsequently underwent dilation and curettage, with pathology confirming degenerated villous tissue.

Her past medical history included a dental procedure two months prior. She denied blood transfusions, other surgeries, or known HBV infection. Obstetric history included gravida 3, para 1, with two induced abortions. Her last menstrual period was December 29, 2016. She denied unsafe sexual contact. Her husband and son were negative for hepatitis B markers, and there was no family history of HBV.

#### Clinical findings

On admission, she was asymptomatic, with the exception of mild scleral icterus.

#### Timeline

Day -6: Hospitalized for missed abortion, HBsAg >250 IU/mL detected. Day -2: Dilation and curettage performed. Day 0 (Admission): HBsAg >250 IU/mL, HBeAg 3.497 S/CO, HBcAb IgM 26.74 S/CO, HBV DNA 2.6×10³ IU/mL, ALT 3657 U/L, AST 1849 U/L, TBil 42.1 μmol/L, DBil 28.5 μmol/L, WBC 6.2×10^9^/L, Hb 119 g/L, PLT 51×10^9^/L. Week 2: HBsAg seroconverted to negative. Day 50: HBcAb IgM became undetectable. Month 7+: Anti-HBs appeared (**Tables 1**, **2**).

**Table 1:** The patient’s hepatitis B serologic markers and liver enzyme levels.

Time	HBsAg (IU/ml)	HBsAb (mIU/ml)	HBeAg (S/CO)	HBeAb (S/CO)	HBcAb (S/CO)	HBcAb IgM (S/CO)	HBV DNA (IU/ml)	ALT (U/L)	AST (U/L)
Day -6	>250(+)	0.00(-)	55.719(+)	2.90(-)	3.42(-)	0.07(-)	-	20	23
Day -1	-	-	-	-	-	-	-	3397	2340
Day 0 (Admission)	>250(+)	2.74(-)	3.497(+)	0.05(-)	0.01(+)	26.74(+)	2.6×10^3^	3657	1849
Day 3	23.10(+)	5.27(-)	0.260(-)	0.01(+)	0.01(+)	27.26(+)	2.6×10^2^	1961	369
Day 10	0.15(-)	1.08(-)	0.299(-)	0.01(+)	0.01(+)	26.97(+)	<100	256	32
Day 24	0.06(-)	0.00(-)	0.225(-)	0.02(+)	0.01(+)	22.63(+)	-	25	19
Day 41	0.03(-)	0.90(-)	0.234(-)	0.01(+)	0.01(+)	18.85(+)	<100		
Day 50	0.01(-)	1.89(-)	0.268(-)	0.01(+)	0.01(+)	0.05(-)	-	17	18
Month 3	0.00(-)	0.00(-)	0.253(-)	0.00(+)	0.01(+)	0.05(-)	-	16	18
Month 7+	0.00(-)	35.47(+)	0.301(-)	0.301(+)	0.01(+)	0.18(-)	-	9	14
Month 11+	0.00(-)	10.51(+)	0.310(-)	0.01(+)	0.01(+)	0.05(-)	-	9	13

**Table 2:** The patient’s complete blood count results.

Time	WBC (10^9^/L)	RBC (10^12^/L)	Hb (g/L)	PLT (10^9^/L)
Day -6	6.2	4.49	129	166
Day 0(Admission)	6.2	4.22	119	51
Day 3	7.1	4.62	135	76
Day 10	6.5	4.21	122	43
Day 12	6.5	4.56	124	62
Day 17	7.5	4.40	122	70
Day 24	6.3	4.31	122	151
Day 50	7.0	4.81	139	197
Month 3	7.7	4.78	136	218
Month 7+	6.3	4.63	133	218
Month 11+	5.9	4.44	124	151

#### Diagnostic assessment

Laboratory tests showed acute hepatocellular injury with elevated ALT/AST and positive HBV markers. Tests for hepatitis A, C, D, and E were negative, as were serologies for cytomegalovirus and Epstein-Barr virus. Autoantibodies (ANA, SLA/LP, anti-LKM) were negative. Abdominal ultrasound showed a hepatic cyst and chronic cholecystitis. Based on clinical, serological, and virological findings, a diagnosis of AHB was made.

#### Therapeutic intervention

She received supportive treatment with liver-protective and enzyme-lowering medications, including compound glycyrrhizin, reduced glutathione, and adenosylmethionine.

#### Follow-up and outcomes

Two weeks after admission, HBsAg became negative. HBcAb IgM became undetectable by day 50, and anti-HBs appeared more than seven months later. HBV DNA clearance was synchronized with HBsAg seroconversion. Liver function normalized and remained stable during one year of follow-up.

#### Patient perspective

During follow-up, the patient reported persistent depressed mood and difficulty concentrating on work, reflecting significant psychological distress despite favorable biomedical recovery.

### Case 2

#### Patient information

A 23-year-old woman at 8 weeks of gestation presented with a two-week history of aversion to greasy food and one week of fatigue. Past medical history was unremarkable; she denied blood transfusions, surgical procedures, or hepatitis B infection. Obstetric history: gravida 0, last menstrual period April 12, 2018. She denied high-risk sexual behavior. Family history was negative for hepatitis B.

#### Clinical findings

On admission, physical examination was unremarkable.

#### Timeline

Day -14: Onset of aversion to greasy food. Day -7: Development of fatigue. Day 0 (Admission): HBsAg 44.45 IU/mL, HBeAg 44.938 S/CO, anti-HBc IgM 2.08 S/CO. ALT 512 U/L, AST 445 U/L, TBil 15 μmol/L. HBV DNA 1.8×10² IU/mL, Intrauterine early pregnancy confirmed by ultrasound. Hospital Day 6: HBsAg became negative, anti-HBs positive. Month 1: HBV DNA undetectable. Month 6: Liver function remained normal during follow-up (**Tables 3**, **4**).

**Table 3:** The patient’s hepatitis B serologic markers and liver enzyme levels.

Time	HBsAg (IU/ml)	HBsAb (mIU/ml)	HBeAg (S/CO)	HBeAb (S/CO)	HBcAb (S/CO)	HBcAb IgM (S/CO)	HBV DNA (IU/ml)	ALT (U/L)	AST (U/L)
Day 0 (Admission)	44.45(+)	0.5(-)	44.938(+)	4.1(-)	0.02(-)	2.08(+)	-	512	445
Day 2	6.35(+)	2.19(-)	4.812(+)	0.59(+)	0.01(+)	5.29(+)	1.8×10^2^	741	422
Day 6	0.02(-)	10.46(+)	0.651(-)	0.31(+)	0.01(+)	5.38(+)	61.4	213	53
Day 10	0.00(-)	12.58(+)	0.460(-)	0.44(+)	0.01(+)	8.09(+)	31.6	77	24
Day 14	-	-	-	-	-	-	-	37	21
Month 1+	0.01(-)	4.49(-)	0.235(-)	0.2(+)	0.01(+)	0.05(-)	<30	21	24
Month 3+	0.00(-)	61.04(+)	0.315(-)	0.01(+)	0.01(+)	0.05(-)	<30	13	20
Month 4+	0.00(-)	62.76(+)	0.292(-)	0.01(+)	0.01(+)	2.03(+)	<30	11	17
Month 6+								12	15

**Table 4:** The patient’s complete blood count results.

Time	WBC (10^9^/L)	RBC (10^12^/L)	Hb (g/L)	PLT (10^9^/L)
Day 0 (Admission)	5.1	4.52	133	158
Day 2	6.0	4.11	123	162
Day 6	5.8	4.14	120	172
Day 10	5.8	4.14	121	179
Day 14	4.1	4.01	118	185
Month 3+	5.7	4.53	132	196
Month 4+	7.3	4.46	124	184

#### Diagnostic assessment

CBC was normal. Serologies for hepatitis A, C, D, and E, and non-hepatotropic viruses were negative. Autoantibody panels were negative. Abdominal ultrasound showed a hyperechoic hepatic nodule consistent with hemangioma. Serum β-hCG was 2057.4 IU/L; gynecological ultrasound confirmed intrauterine pregnancy. Diagnosis: AHB with early intrauterine pregnancy.

#### Therapeutic intervention

Supportive treatment was provided with liver-protective therapy (compound glycyrrhizin) without antiviral agents.

#### Follow-up and outcomes

HBsAg clearance occurred by hospital day 6, and HBV DNA became undetectable within one month. Liver function remained normal during six months of follow-up.

#### Patient perspective

Despite favorable clinical improvement, the patient persistently expressed marked anxiety regarding liver failure and potential fetal harm. Even after repeated reassurance by physicians, her anxiety remained high throughout hospitalization and follow-up.

### Case 3

#### Patient information

A 25-year-old woman at 10 weeks of gestation presented with one week of dizziness and fatigue, accompanied by low-grade fever and a generalized pruritic rash for one day. Past medical history was unremarkable; she denied transfusions, surgery, or prior HBV infection. Obstetric history: gravida 0, last menstrual period March 20, 2018. She denied high-risk sexual behavior. Family history was negative for hepatitis B. Pre-pregnancy serological screening confirmed HBsAg negativity.

#### Clinical findings

On admission, she exhibited mild scleral icterus and scattered erythematous skin eruptions on the upper limbs.

#### Timeline

Day -7: Onset of dizziness and fatigue. Day -1: Development of low-grade fever and generalized rash. Day 0 (Admission): ALT 1798 U/L, AST 1508 U/L, TBil 51.8 μmol/L. Day 1: HBsAg >250 IU/mL, HBeAg 8.666 S/CO, anti-HBc IgM 22.27 S/CO. HBV DNA 7.8×10^6^ IU/mL, ALT 1136 U/L, AST 434 U/L. Coagulation: mildly prolonged PT, no encephalopathy. CBC normal. Abdominal ultrasound: slight increase in hepatic echogenicity. Hospital Day 5: HBsAg negative, anti-HBs positive. Hospital Day 15: HBV DNA declined to 1.9×10³ IU/mL with marked biochemical improvement. Post-discharge: Patient did not return for further follow-up (**Tables 5**, **6**).

**Table 5:** The patient’s hepatitis B serologic markers and liver enzyme levels.

Time	HBsAg (IU/ml)	HBsAb (mIU/ml)	HBeAg (S/CO)	HBeAb (S/CO)	HBcAb (S/CO)	HBcAb IgM (S/CO)	HBV DNA (IU/ml)	ALT (U/L)	AST (U/L)
Day 0 (Admission)	-	-	-	-	-	-	-	1798	1508
Day 1	>250(+)	9.05(-)	8.666(+)	0.04(+)	0.01(+)	22.27(+)	7.8×10^6^	1136	434
Day 5	0.01(-)	26.04(+)	0.43(-)	0.01(+)	0.01(+)	25.36(+)	8.2×10^3^	446	96
Day 8	-	-	-	-	-	-	-	215	34
Day 11	-	-	-	-	-	-	-	80	25
Day 15	0.00(-)	1.71(-)	0.354(-)	0.04(+)	0.01(+)	25.34(+)	1.9×10^3^	32	29
Day 19	-	-	-	-	-	-	-	26	32

**Table 6:** The patient’s complete blood count results.

Time	WBC (10^9^/L)	RBC (10^12^/L)	Hb (g/L)	PLT (10^9^/L)
Day 1	7.3	4.12	124	205
Day 5	13.5	4.05	122	235
Day 8	9.7	3.99	120	254
Day 11	8.9	3.87	115	291
Day 15	9.9	3.97	118	336
Day 19	9.4	3.87	116	372

HBsAg, hepatitis B surface antigen; HBsAb, hepatitis B surface antibody; HBeAg, hepatitis B e antigen; HBeAb, hepatitis B e antibody; HBcAb, hepatitis B core antibody; HBcAb IgM, immunoglobulin M antibody against hepatitis B core antigen; HBV DNA, hepatitis B virus deoxyribibonucleic acid; ALT, alanine aminotransferase; AST, aspartate aminotransferase; WBC, white blood cell; RBC, red blood cell; Hb, hemoglobin; PLT, platelet.

#### Diagnostic assessment

Serologies for hepatitis A, C, D, and E viruses were negative. Non-hepatotropic viral panels were negative. Autoantibody screening revealed anti-mitochondrial antibody M2 positivity, raising the possibility of primary biliary cholangitis (PBC), though no follow-up evaluation was completed. Gynecological ultrasound confirmed a viable intrauterine pregnancy. Based on acute onset and serology, she was diagnosed with AHB with intrauterine pregnancy.

#### Therapeutic intervention

She received supportive therapy with liver-protective, enzyme-lowering, cholagogic, and antijaundice agents (compound glycyrrhizin, polyenylphosphatidylcholine, and adenosylmethionine).

#### Follow-up and outcomes

During hospitalization, the patient demonstrated rapid clinical and virological improvement. However, she did not return for post-discharge follow-up, limiting assessment of long-term outcomes or further evaluation of possible PBC.

#### Patient perspective

The patient reported profound anxiety about her prognosis and fetal health. She repeatedly sought reassurance from physicians regarding potential complications, vertical transmission risk, and long-term impacts, despite favorable virological and biochemical responses. Her persistent psychological distress underscored the significant mental health burden associated with perinatal HBV infection.

## Discussion

AHB during pregnancy, though uncommon, is a clinically important condition that requires multidisciplinary management. This case series presents a unique perspective on the management and implications of AHB during pregnancy.

In most adults with intact immunity, AHB is a self-limited condition, with spontaneous viral clearance achieved in over 90% of cases through a robust T cell-mediated immune response (12). From an immunological perspective, the ability of AHB patients to clear infection is largely driven by vigorous CD8+ T cell activation. Markers such as CD69 and CD178 on CD8+ lymphocytes indicate enhanced cytotoxic and apoptotic activity, explaining why most AHB patients, unlike chronic carriers, succeed in eliminating HBV (13–15). However, pregnancy creates a unique immune environment where the mother’s cellular immune response is temporarily suppressed. This adaptation helps her body tolerate the developing fetus, which expresses foreign (paternal) antigens. Elevated levels of progesterone, estrogen, and human chorionic gonadotropin exert inhibitory effects on T cell function, resulting in weakened HBV-specific CD8+ responses compared with non-pregnant individuals (16). Consequently, pregnant women exposed to HBV may be at greater risk of progression to chronic infection. Furthermore, the clearance of HBsAg and seroconversion may be delayed in pregnant women with AHB compared to non-pregnant individuals with the same condition (17).

In the first patient analyzed, who was in the latent period following AHB infection, the dynamic changes of HBsAg viral markers during the AHB could be fully observed. HBcAb-IgM appeared 7 days later than HBsAg and HBeAg, persisted for 50 days, and HBsAg disappeared after 16 days. HBsAb appeared at 7 months, HBeAg disappeared on day 10 of the disease course, accompanied by the appearance of HBeAb positivity. The duration of HBV DNA was synchronized with HBsAg, disappearing at 16 days of the disease course. ALT and AST levels returned to normal within 1 month. During the disease course, this patient developed thrombocytopenia, with the lowest platelet count detected at 43×10^9^/L. As the patient showed no significant bleeding, no intervention was taken, and close monitoring was maintained. The thrombocytopenia persisted for 24 days and subsequently resolved. It is speculated that the thrombocytopenia may be associated with the onset of HBV infection. In the second patient analyzed, who was positive for HBsAg on admission but seroconverted to negative on the sixth day of hospitalization. Given the relatively low HBV DNA level, it is considered that partial spontaneous viral clearance had already occurred prior to admission. Following HBsAg seroconversion, transitions in HBsAb and HBeAg were observed shortly afterward. HBV DNA became undetectable one month later than HBsAg, and HBcAb IgM disappeared within 1 month after admission. AST and ALT levels returned to normal by day 14. In the last patient analyzed, who’s HBsAg seroconverted to negative on the fifth day after admission. Unfortunately, due to poor compliance, we were unable to monitor the point at which HBV DNA levels fell below the detectable limit. Additionally, the patient tested positive for the anti-mitochondrial antibody M2 subtype, suggesting that the elevated bilirubin and abnormal liver enzymes may also be related to PBC. However, no further follow-up or treatment could be provided.

In these cases, the patient cleared the virus naturally without antiviral treatment, as shown by the rapid loss of HBsAg, illustrating the favorable outcome of AHB. Although our patients achieved rapid virological clearance, they experienced significant anxiety regarding their own and fetal health. In many societies, particularly in China where HBV is common, there is a strong social stigma against the virus. This stigma significantly increases anxiety for patients. Although acute HBV infection in early pregnancy does not increase the risk of birth defects or miscarriage, the virus can be passed to the newborn in about 10% of cases. On one hand, some experts advocate for a conservative approach without antiviral therapy, citing evidence that most patients experience a sharp decline in HBV DNA within one month and approximately 95% of adults achieve spontaneous recovery. Furthermore, long-term use of medication may lead to drug-resistant mutations. A study found that drug-resistant mutations were detected in 32.66% of hepatitis B patients (18). On the other hand, proponents of antiviral intervention argue that early treatment may enhance viral clearance, reduce HBsAg and HBV DNA levels, prevent chronic infection, and ultimately reduce the risk of hepatocellular carcinoma. Notably, several professional organizations recommend antiviral therapy at the 28th week of pregnancy if the maternal HBV DNA is >200,000IU/ml, as a conservative choice to minimize vertical HBV transmission (19, 20). For breastfed infants receiving birth-dose vaccine and immune globulin, the risk of HBV infection is minimal (21). But these uncertainties may overwhelm pregnant women.

As all three patients achieved HBsAg clearance, with two reaching virological cure, no adverse virological responses were observed. It is therefore important to consider potential outcomes in less favorable scenarios. Pregnant women have reduced immune clearance, and persistence of HBsAg beyond six months indicates progression to chronic hepatitis B, which increases maternal risks of fibrosis, cirrhosis, and hepatocellular carcinoma, as well as pregnancy complications such as gestational hypertension and diabetes. High maternal viral load, particularly >200,000 IU/mL in late pregnancy, also markedly increases the risk of mother-to-child transmission (22, 23). Without vaccination, 90% of newborns exposed to HBV will develop chronic infection, compared to 5-10% of adults exposed to HBV (24).

A comprehensive review on viral hepatitis in pregnancy noted that CHB is characterized by persistent viral replication. Maternal HBV DNA levels > 2×10^5^ IU/mL are associated with a 10%-15% mother-to-child transmission (MTCT) risk despite standard immunoprophylaxis (25). In contrast, a hepatologist’s perspective on hepatitis B management in pregnancy stated that AHB in pregnancy has variable MTCT risks: first - trimester AHB carries a 10% transmission rate, while third - trimester infection raises this to 60% without intervention. However, rapid viral clearance can eliminate transmission risk (26). Management strategies also differ: AHB in pregnancy mainly requires supportive care, with antiviral intervention reserved for severe cases. On the other hand, CHB management focuses on long-term viral suppression to prevent postpartum flares and MTCT. A review on HBV diagnosis and treatment further emphasized that for CHB in pregnancy, antiviral therapy aims to suppress viral replication to reduce MTCT, while AHB treatment is more focused on addressing acute liver injury (27). Notably, while the review on viral hepatitis in pregnancy mentioned that CHB in pregnancy is linked to increased risks of gestational diabetes and antepartum hemorrhage, the impact of AHB on obstetric outcomes remains understudied. A study suggest no elevated maternal mortality in AHB during pregnancy but potential associations with preterm birth and low birth weight in cases of severe liver injury (26).

According to a 2023 global review, rates of anxiety during pregnancy have been gradually rising (28, 29). Antenatal anxiety and depression have been associated with preeclampsia, premature birth, low birth weight, neonatal infections, and impaired cognitive development in offspring (30, 31). Therefore, it is essential to incorporate psychosocial assessment into the comprehensive management of pregnant women infected with HBV. Although our cases highlight the psychological distress associated with acute HBV infection in pregnancy, systematic mental health protocols were not implemented during their clinical care. To address this, we propose a scalable framework for integrating mental health screening into HBV perinatal care. This includes: routine use of validated screening tools such as the Generalized Anxiety Disorder-7or the Edinburgh Postnatal Depression Scale; stratified management according to severity, with mild cases receiving enhanced counseling and reassurance, and moderate-to-severe cases referred for professional psychological support; establishing multidisciplinary collaboration among hepatologists, obstetricians, and mental health specialists; and incorporating mental health assessment into postpartum follow-up. Such an approach could help reduce anxiety, improve treatment adherence.

## Limitations

This study has several limitations. First, its retrospective nature precluded the use of standardized psychometric instruments, so anxiety was assessed qualitatively, which may limit objectivity. Second, the sample size was very small and lacked a control group, making it difficult to determine whether the observed psychological distress was specific to acute HBV in pregnancy or a common perinatal phenomenon. Third, all patients showed favorable virological outcomes, which may introduce selection bias and limit generalizability to women with poorer responses or risk of chronicity. Finally, one patient was lost to follow-up, restricting assessment of long-term outcomes. These cases nevertheless provide valuable clinical insights and highlight the need for prospective studies with larger cohorts, control groups, validated psychometric tools, and extended follow-up.

In conclusion, AHB in pregnancy is usually self-limited with a good prognosis, but may cause psychological distress affecting reproductive decisions. A patient-centered approach is essential for better clinical and quality-of-life outcomes, bridging hepatology and mental health helps support these women.

## Data Availability

The original contributions presented in the study are included in the article/supplementary material. Further inquiries can be directed to the corresponding author.
